# Soft-sensor model development for CHO growth/production, intracellular metabolite, and glycan predictions

**DOI:** 10.3389/fmolb.2024.1441885

**Published:** 2024-10-22

**Authors:** George Liang, Sha Sha, Zhao Wang, Huolong Liu, Seongkyu Yoon

**Affiliations:** Department of Chemical Engineering, University of Massachusetts Lowell, Lowell, MA, United States

**Keywords:** fed-batch bioprocess, Michaelis-Menten, monod kinetics, machine learning, datadriven, glycosylation

## Abstract

Efficaciously assessing product quality remains time- and resource-intensive. Online Process Analytical Technologies (PATs), encompassing real-time monitoring tools and soft-sensor models, are indispensable for understanding process effects and real-time product quality. This research study evaluated three modeling approaches for predicting CHO cell growth and production, metabolites (extracellular, nucleotide sugar donors (NSD) and glycan profiles): Mechanistic based on first principle Michaelis-Menten kinetics (MMK), data-driven orthogonal partial least square (OPLS) and neural network machine learning (NN). Our experimental design involved galactose-fed batch cultures. MMK excelled in predicting growth and production, demonstrating its reliability in these aspects and reducing the data burden by requiring fewer inputs. However, it was less precise in simulating glycan profiles and intracellular metabolite trends. In contrast, NN and OPLS performed better for predicting precise glycan compositions but displayed shortcomings in accurately predicting growth and production. We utilized time in the training set to address NN and OPLS extrapolation challenges. OPLS and NN models demanded more extensive inputs with similar intracellular metabolite trend prediction. However, there was a significant reduction in time required to develop these two models. The guidance presented here can provide valuable insight into rapid development and application of soft-sensor models with PATs for ipurposes. Therefore, we examined three model typesmproving real-time product CHO therapeutic product quality. Coupled with emerging -omics technologies, NN and OPLS will benefit from massive data availability, and we foresee more robust prediction models that can be advantageous to kinetic or partial-kinetic (hybrid) models.

## 1 Introduction

Monoclonal antibodies (mAbs) are therapeutic proteins with wide-ranging applications (cancer, arthritis, multiple sclerosis, heart disease, etc.). Their efficacy, safety, solubility, and pharmacokinetics/pharmacodynamics are partially dictated by post-translational modifications (PTMs). N-linked glycosylation is a vital PTM that often serves as a manufacturing product quality attribute (PQA). Feeding strategies and genetic engineering are common approaches to modulating glycan profiles (Niu et al., 2018; Sha Sha and Yoon, 2019) and improve mAb glycosylation profile heterogeneity (Chen et al., 2018; Bingyu et al., 2024; Beck and Liu, 2019). Assessing this micro-heterogeneity utilizes analytical tools such as (1) pH gradient cation exchange chromatography, in which charge heterogeneity is determined from cell culture supernatant without any purification steps (Sissolak et al., 2019), and (2) rapid labeling techniques coupled with mass-spectroscopy (Kameyama et al., 2018).

PATs can provide real-time monitoring and control for PQAs through feed or media composition. Recent PAT advances include utilizing an online sequential-injection-based system coupled with rapid labeling techniques (N-GLYcanyzer) to integrate mAb sampling and preparation for glycan analysis with high-performance liquid chromatography (HPLC) (Gyorgypal and Chundawat, 2022) and rapid detection utilizing lectin-based assays to target specific glycan residues (Tulin et al., 2021). Saunders et al. recently developed a carbohydrate-sensing microsphere that simultaneously detects multiple orthogonal glycosylation features for rapid identification (GlycoSense) (Saunders et al., 2023). Predictive models can complement PATs for monitoring/controlling mAb glycan profile homogeneity, helping control feed and media conditions to achieve a specific target glycan profile and providing insight into glycan synthesis and degradation machinery that underlie dysregulated glycosylation (Krambeck et al., 2017; Coral et al., 2021; Li et al., 2022). Soft-sensor models (kinetic, machine learning, hybrid) are emerging to help predict glycan profiles better. Most often, machine learning and data-driven models cannot extrapolate beyond the dataset for prediction. Hybrid models can mitigate certain aspects of this “black box” by providing a conceptual biological system for prediction (i.e., intracellular metabolites) and extrapolation. However, we observed that machine learning and data-driven models can, in fact, extrapolate and provide significant biological system knowledge, circumventing the need to provide intracellular metabolites for accurate glycan predictions.

Several models have demonstrated their capabilities to monitor and predict glycosylation profiles, with a few having linked metabolites and nucleotide sugar transports to the Golgi apparatus for mAb high-throughput profiling (Jedrzejewski et al., 2014; Villiger et al., 2016). Others have considered how intracellular processes determining antibody Fc-glycosylation impact mAb production and PQAs with mild hypothermia (Sou et al., 2017). By combining two kinetic modules (cell metabolism and NSD synthesis), a hybrid-artificial neural network model (HyGlycoM) utilizes the kinetic output of its neural network model to improve glycan profile prediction (Pavlos and Cleo, 2020). GlyCompare™ is another example that allows intermediate glycan accounting to connect measured glycans to provide future glycan structure prediction extrapolation (Bao et al., 2021). SweetNet transfers a glycan into a graphical representation and learns the similarity of glycans for predicting their organismal phenotypic and environmental functions (monosaccharides and linkages of glycans) and retains valuable structural information (Rebekka et al., 2021). Other models have been used to investigate stereoselectivity for glycan biosynthesis, glycan-mediated host-microbe interactions, predict lectin to glycan binding specificities, glycan functions, and immunogenicity (Li et al., 2022; Moon et al., 2021; Athanasios et al., 2021; Lundstrøm et al., 2022; Bojar et al., 2022). Besides glycosylation predictions, models have been used for cellular growth and production improvements. Clarke et al. combined transcriptomic gene data to predict cell-line specific productivity with a PLS model to achieve a 4.44 pg/cell/day root mean squared error in cross-model validation (RMSE^CMV^) (Colin et al., 2011). Yahia et al. developed an empirical metabolic model connecting extracellular metabolic fluxes with cellular growth and product formation with mixed Monod-inhibition type kinetics to describe integral viable cell density (IVCD) and mAb production to assess new feeding strategies and operating conditions (Ben Yahia et al., 2021). Selvarasu et al. developed a PLS model correlating amino acids (AA) with VCD and productivity (Selvarasu et al., 2010).

These models can be classified as mechanistic (kinetic or stoichiometric), data-driven, and/or machine-learning. Kinetic models provide additional information to help optimize the cell culture process and cell metabolism; however, they require a better understanding of the underlying CHO machinery mechanism to enhance the model’s prediction. Due to limited regulatory information, most mechanistic pathways are not involved in the current *in silico* models. Although kinetic models are built to portray some biological function, they often make specific assumptions, rendering them less adaptable to different cultural conditions and requiring extensive parameter estimation and optimization (Apostolos and Ioscani, 2021). Their lack of standardization impedes merging smaller models into larger ones. One way modelers face uncertainty challenges are by including it. Mechanistic modeling of the CHO biological system is usually an under-constrained problem that has more variables than observations, increasing the models’ degree of freedom (Coral et al., 2021; Ben Yahia et al., 2021; Almquist et al., 2014; Arigoni-Affolter et al., 2019; Zhang et al., 2020; Hong et al., 2022).

OPLS and NN models are advantageous in prediction when there is a need for more understanding of the complex physical mechanisms of the biological system. However, accurate predictions typically require large data sets, equating to intensive resources and high costs. These models are typically incapable of extrapolation outside the trained conditions, and glycosylation data lacks standardization and reliability across different research groups (Coral et al., 2021; Li et al., 2022; Apostolos and Ioscani, 2021; Hong et al., 2022). Therefore, these models can provide unreliable prediction performance when unseen glycans or results arise. Overall, glycan modeling has multiple intricate and diverse modeling approaches that merit examination for quality purposes. Therefore, we examined three model types (kinetic (MMK), data-driven (OPLS), and neural network machine learning (NN)) to distinguish the advantages (and disadvantages) for each approach, focusing on 1) growth and production, 2) intracellular metabolites (NSD) and 3) glycan prediction and extrapolation.

## 2 Materials and methods

### 2.1 Cell culture

The cell culture work was reported previously (Sha Sha and Yoon, 2019). In brief, a glutamine synthetase (GS) CHO cell line produced an immunoglobulin (IgG) biosimilar protein to Adalimumab with the experimental design (Table 1) considering different galactose concentrations (0 or 25 mM) and feeding times (72 and 120 h).

**TABLE 1:** Design of experiment for GS CHO with galactose supplementation at different cell culture process time (n ≥ 2).

Condition	Gal concentration (mM)	Time (hr)
Control	n/a	n/a
A	25	72
B	25	120
Validation^a^	25	72 and 120

^a^ Biological cell culture replicates were conducted. In addition, the average of these two runs were used to create an average experimental condition for model validation (n = 3).

#### 2.1.1 Mammalian cell culture condition

GS CHO cells were thawed and expanded in a humidified shaking incubator at 37°C and 5% CO_2_. When the cells expanded to 4 × 10^6^ cells/mL ± 0.5 × 10^6^ cells/mL, the cells were inoculated into eight flasks (70 mL working volume in a 250 mL shake flask) at (0.3 ± 0.15 × 10^6^ cells/mL) following the design of experiment (DoE) shown in Table 1. The CD FortiCHO™ Medium (Thermo Fisher Scientific, Waltham, MA) was used as the basal media in all the experiments. Briefly, a control set (n = 2) was cultured without galactose. Metabolites were collected every 12–24 h from Day 0 to Day 6 (10 data points). Nucleotide sugar samples were collected every 12 h from Day 2.5 to Day 5.5 (2-7 data points). Glycan samples were collected roughly every 24 h from Day 3 to Day 6 (3 data points). Set A (n = 2) were fed 25 mM galactose on Day 3. Metabolites were collected every 12–24 h from Day 0 to Day 9 (13 data points). Nucleotide sugar samples were collected every 12 h from Day 2.5 to Day 9 (3–10 data points). Glycan samples were collected roughly every 24 h from Day 3 to Day 7 (3-5 data points). Galactose measurements were collected every 24 h from Day 3 to Day 6.5 (5 data points). Set B (n = 2) were fed with 25 mM galactose on Day 5. Set B collected the same amount of data as Set A. The validation set (n = 2) were fed with 25 mM galactose twice on Day 3 and Day 5, respectively. Metabolites were collected every 12–24 h from Day 0 to Day 12 (18 data points). Nucleotide sugar samples were collected every 12 h from Day 2.5 to Day 9 (3–12 data points). Glycan samples were collected from Day 3 to Day 8 and analyzed at Day 3, 4, 6, 7 and 8. Galactose measurements were collected every 12–24 h from Day 3 to Day 8 (7 data points). Furthermore, in the validation set, the two biological shake flasks were averaged to produce a “theoretical pooled” biological shake flask to ascertain any statistical differences.

#### 2.1.2 Antibody purification

Pierce^TM^ magnetic protein A/G agarose beads (ThermoFisher, Waltham, MA) were used for antibody purification. Briefly, 20 µL of bead slurry was used. 230 μL of 50 mM Sodium Phosphate pH 7.0 was used to pre-condition the beads. The slurry was placed onto a magnetic stand and supernatants were removed. This step was repeated a second time prior to sample addition. The samples were then incubated on an orbital shaker plate for 15 min at 350 rpm. The supernatant was then removed again from the magnetic stand. The samples were then washed with sodium phosphate and removed with the magnetic stand; repeated a second time. Then repeated with MilliQ water (MilliporeSigma, Burlington, MA). The samples were eluted with 100 mM sodium phosphate pH 2.5 and incubated on the orbital shaker for 10 min at 350 rpm. The samples are then placed into the magnetic stand and the solutions were collected and neutralized with 500 mM sodium phosphate pH 8.0. The final amount of protein recovered was analyzed with a nanodrop (ThermoFisher, Waltham, MA).

### 2.2 Analysis of N-linked glycan mAb produced under different supplemental conditions

#### 2.2.1 N-linked glycan isolation

The N-glycans were isolated using a kit supplied by New England Biolabs (NEB, Ipswich, MA). Samples containing 20 µg mAb were first denatured using 1x denaturation buffer for 10 min at 100 °C. Then cooled on ice for 1 min followed by release of the glycans by treating with PNGaseF enzyme at 37°C for 2 h. As per the manufacturer recommended protocol, we used the 1x reaction buffer and 1% NP-40 in the de-glycosylation procedure.

#### 2.2.2 Labeling N-glycans with 2-AB and cleaning

The N-glycan samples were derivatized with 0.35 M 2-AB (MilliporeSigma, St. Louis, MO, USA) and 1 M Borane -2-methylpyridine complex 95% (MilliporeSigma, Burlington, MA) dissolved in a mixture of 70% DMSO (MilliporeSigma, Burlington, MA) and 30% acetic acid (MilliporeSigma, Burlington, MA), for 2 h at 65°C. The labeled N-glycan samples were then cleaned to remove excess dye by using 100 mg/mL HyperSep Diol SPE Cartridges (ThermoFisher, Waltham, MA). The columns were prewashed with MilliQ water. Then primed 4 times with Acetonitrile (MilliporeSigma, Burlington, MA). Samples were diluted 1:9 with Acetonitrile and loaded into the column. It was then further washed with Acetonitrile. The samples were eluted with MilliQ water. The purified labeled N-glycans were dried for 2 h in a Speed Vac freeze-dryer (ThermoFisher, Waltham, MA) and resuspended in 15 µL of Milli-Q water.

#### 2.2.3 N-linked glycan characterization by HPLC analysis

Analysis of cleaned 2-AB labeled N-glycan samples were performed using a previously published method (Sha Sha and Yoon, 2019). This method involves a buffer A of pH 4.5 made up of 100 mM ammonium formate (Millipore Sigma, Burlington, MA), and a buffer B of 100% acetonitrile (Millipore Sigma, Burlington, MA). Separation of N-glycans was performed on a Acquity UPLC BEH amide Glycan column - 2.1 mm × 50 mm, 1.7 µm (Waters, Milford, MA). The column was pre-equilibrated with 25% buffer A at 50°C column temperature. 2 μL of the N-glycan sample was injected and the elution was performed with the following conditions: the gradient included a decrease of buffer B from 70% to 65% over 20 min and then 65%–60% over 5 min. The wavelength of excitation was 350 nm, and the emission was 420 nm. HPLC analysis was performed on an Agilent 1,100 high pressure liquid chromatography system (Agilent, Santa Clara, CA).

### 2.3 Analysis of cell culture and metabolites

Cell count and viability was analyzed using Cedex Hires (Roche Life Science, Indianapolis, IN). Extracellular metabolites (glucose, lactate, ammonium, glutamine, and glutamate) were measured with a Nova Flex I (Nova Biomedical, Waltham, MA). mAb titer was analyzed with an Agilent 1100 HPLC as previously described (Sha Sha and Yoon, 2019). Intracellular metabolites were measured by a HPLC as previously described (Sha Sha and Yoon, 2019). Galactose measurements were measured by a HPLC-RID system as previously described (Sha Sha and Yoon, 2019).

## 3 Model development

The work’s focus is to establish three different types of models: a mechanistic kinetic model (MMK), a data-driven model (OPLS), and a machine learning neural networks model (NN) and evaluate their capability for prediction in 1) growth and production, 2) intracellular metabolites, and 3) glycan profile with a galactose and time-dependent fed case study. A total of 108 distinct data points were used for model training: reference Appendix -Supplementary Table SC1.

### 3.1 Mechanistic kinetic model (MMK)

The kinetic-based mechanistic modeling framework aims to estimate the intracellular nucleotide sugar concentrations based on extracellular glucose and glutamine levels and galactose, with a goal of predicting the impact of feeding strategies on glycan distribution. It comprises an unstructured cell growth model, mAb production and kinetic models of nucleotide and nucleotide sugar synthesis, and an N-linked glycosylation maturation model. The platform used to build the model is gPROMS Formulated Products 2.3.0 (Siemens Process Systems, Lowell, MA). This kinetic model is modified based on Jedrzejewski et al. glycan model framework (Jedrzejewski et al., 2014). The following assumptions were made for MMK: (1) saturation of enzyme availability and instantaneous reaction, (2) steady state is achieved, (3) single substrate Michaelis-Menten kinetics are assumed to be uni-uni enzyme kinetics, whereas multiple substrate Michaelis-Menten kinetics are assumed to be Bi-ternary complex, (4) transport of nucleotide sugar donor was assumed to have a constant fluxed transport, (5) glycan byproduct formation is assumed to be negligible. The modeling equations with detailed modifications are listed in the Supplementary Appendix - B.I. In brief, changes were made to include galactose kinetic equations accounting for galactose supplementation. Michaelis Menten kinetic reaction equations were simplified from single substrate uni-uni enzyme kinetics, random order bi-bi enzyme kinetics, ordered bi-bi enzyme kinetics, ping-pong bi-bi enzyme kinetics, and ping-pong ter-ter enzyme kinetics to just single substrate uni-uni enzyme kinetics, due to the progression of glycan maturation being solely formed from the precursor glycan. The model simulates the t_0_ metabolite experimental data points with a theoretical t_0_ for NSD and glycan profile. The theoretical values were based on collected data on t_3_. The model was simulated forward, making feeding adjustments while necessary with a time schedule that dictates when galactose was fed and when sampling was conducted to account for volume change. gPROMs used maximum likelihood estimation to define the kinetic parameter values, which used teacher forcing to minimize the SSE. The kinetic model was trained and validated with experiments (Table 1).

### 3.2 Data-driven multivariant (OPLS) model

The commercial SIMCA (Version 18.0, Sartorius, Cambridge, MA) software is used to generate a batch level orthogonal partial least square (OPLS) model. OPLS is a data driven model that decomposes the predictor X variable into two parts: one that is linearly related to the response variable Y, and second part orthogonal to the first that is not linearly predictive of Y (Vajargah et al., 2012). This method is supervised both in the sense that a linear prediction of Y is obtained, and in that the Y values are required to fix the decomposition of X. The following assumptions were made for OPLS: (1) each data point is assumed to have reached steady state, (2) assumed all data have the same weight, (3) assumed there are no influential outliers in the dataset. The model was trained (Control, Set A, Set B) and validated based on experiments as presented in Table 1. From the given dataset, factor variables were defined as the extracellular metabolites including glucose, glutamine, ammonia, lactate, glutamate, and galactose. The response variables were defined as glycans (G0F, G1F, and G2F), intracellular nucleotide sugar donors (NSD) (UDP-Gal, UDP-Glc, UDP-GalNAc, and UDP-GlcNAc), viable cell density (VCD) and mAb yield. The number of principal components were automatically defined by the software and chosen as 5 with an R2X (cum) of 0.973 and a R2Y (cum) of 0.591 and a Q2 (cum) of 0.34 (Supplementary Figure SC1). To ensure the model was not overparameterized, different number of components were assessed to observe the trend of the expected response variable and five components were demonstrated to be able to generate a decent glycan prediction trend. In a separate model, intracellular NSD was also used as a factor variable. However, the observed glycan profile trend was inaccurately represented by the predictions (Supplementary Figure SC2). Hence, that model was not used for comparison.

### 3.3 Neural network machine learning (NN) model

**FIGURE 1:**
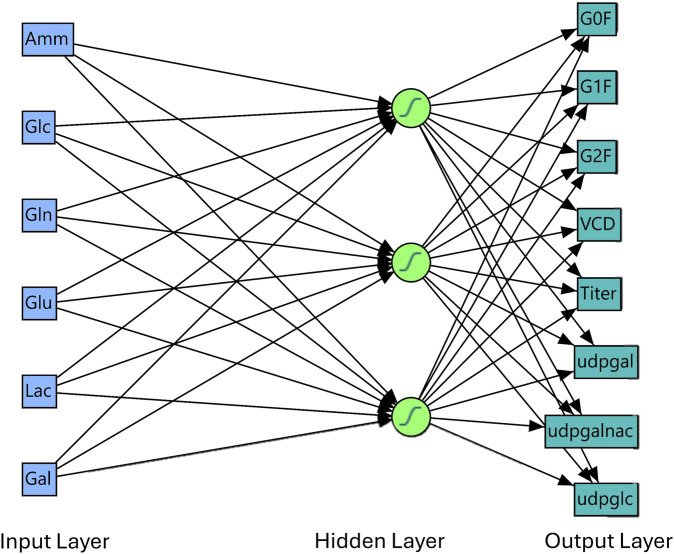
Neural network model Network and Layer. From Figure 1, model consists of an input layer (extracellular metabolites), a hidden layer and an output layer representing our target variables of interest for prediction (glycans, growth, production, and intracellular metabolites). Model overfit was determined by comparing the R^2^ and root average square error (RASE) between the validation and training dataset (Supplementary Table SC2). There was no overfit of the model for each of the output variables.

The platform used to develop the NN model was from commercial software JMP® (Student Edition), version 17.2.0 (Cary, NC). The network and layers were constructed as shown in Figure 1. In brief, the model consists of 3 layers: where extracellular metabolite measurements were used as an input layer, and a tanh activation function hidden layer is used to extract and process the input layers for an output response prediction for a total parameter count of 6⋅3+3+3⋅8+8=53 . The following assumptions were made for NN: (1) each data point is assumed to have reached steady state, (2) neurons are arranged in layers and sequentially arranged, (3) there are no interaction between each neuron within the same layer, (4) inputs enters the network from the input layer and passes through the output layer and are densely connected, (5) same hidden layers have the same activation function, (6) same weight is assumed for all input layers, and (7) same bias is assumed for all hidden layers. The model uses loglikelihood and minimizes the squared sum error (SSE). The fitting algorithm consists of an outer loop that optimizes the penalty parameter and an inner step where the objective function, the likelihood plus penalty function, is optimized using a quasi-Newton method, BFGS, for a particular value of the penalty parameter. In the beginning, normally distributed random starting values (1,234 points) are generated, and the penalty parameter is set to zero. After this initial fit, a nonzero candidate value of the penalty parameter is then chosen, and a univariate line search on the penalty parameter is undertaken. Over the course of this search, the model whose parameters led to the best value of the likelihood on the training set is the one that is reported by the platform. As the BFGS iterations proceed, the value of the likelihood function of the model on the validation data is monitored. When the cross-validation likelihood is no longer improving, the BFGS algorithm will terminate. Zero random holdback of the data was defined to train the model utilizing the experimental datasets: Control, Set A, Set B as shown in Table 1. In the model, the validation datasets were excluded during model training and used for validation only. Model overfit was determined by comparing the R^2^ and root average square error (RASE) between the validation and training dataset (Supplementary Table SC2). There was no overfit of the model for each of the output variables. In addition, the software includes a NN profiler package that can be utilized to understand prediction trends and correlations between variables.

### 3.4 Statistical analyses

**FIGURE 2:**
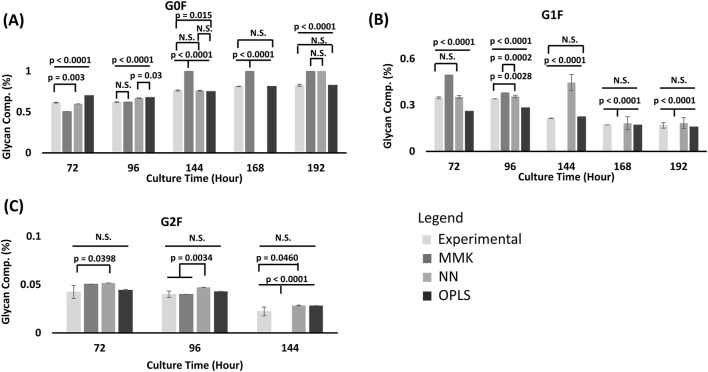
Glycan model validation comparison [Galactose fed at 72 & 120 h]. From **(A)** G0F Glycan; **(B)** G1F Glycan; **(C)** G2F Glycan species prediction from MMK, OPLS and NN model was compared to the experimental (EXP) results at different time periods (72, 96, 144, 168, 192 h) from condition D in Table 1; with double galactose feed (25 mM) at 72 and 120 h. ^1^Glycan composition (%) is the area under the curve (AUC) and normalized to the total sum of the AUC from all glycan peaks (G0F, G1F, and G2F). The standard error bars indicate the standard deviation simulated from the different model output (n = 3). Comparison of mean differences were performed by ANOVA, followed by a Tukey’s HSD mean comparison. ^2^NN model for G0F at 168 h was not predicted. There was no value output from the model. But looking at the trend at the 192 h, it is most likely going to produce a *p* < 0.0001. Which defines that time point as significantly different than the experimental results. ^3^Significant difference is observed when comparisons have a *p*-value ≤ 0.05.

Quantitative differences in the simulated and experimental results were evaluated by analysis of variance (ANOVA) of the integrated peak area (%) values of N-glycan types, VCD, titer and intracellular NSDs. ANOVA was performed on glycan peak area (%) values of the samples (n = 3) corresponding to each of the process simulated culture condition levels at single culture time points followed by comparison of means by Tukey’s HSD (Figure 2 and Supplementary Table SC3). VCD, titer (Table 2, 3) and intracellular NSD (Table 4) were assessed by considering the whole culture duration. Furthermore, a profiler analysis was assessed as well to understand relationships and prediction outcomes observed between the inputs and output variables (Supplementary Figure SC3). For these statistical analyses we used the software JMP® (Student Edition), version 17.2.0 (Cary, NC).

**TABLE 2:** Tukey’s HSD mean comparison between model for VCD.

Level	- Level	Difference^b^	Std err Dif	Lower CL	Upper CL	*p*-value^a^
Exp	Neural	42465.40	10877.79	14272.3	70658.52	0.0008
Kinetic	Neural	39478.57	10877.79	11285.4	67671.69	0.0021
OPLS	Neural	27064.04	10877.79	−1129.1	55257.17	0.0650
Exp	OPLS	15401.35	9474.43	−9154.5	39957.24	0.3667
Kinetic	OPLS	12414.52	9474.43	−12141.4	36970.41	0.5575
Exp	Kinetic	2986.83	9474.43	−21569.1	27542.72	0.9891

^a^ A *p*-value ≤ 0.05 considers the difference significant between the compared 2 groups, either model-model or model-experimental results.

^b^ This difference indicates the mean differences of the triplicate runs. On average, there are 108 different data points utilized to train the model, and validated with.

**TABLE 3:** Tukey’s HSD mean comparison between model for a Titer overview.

Level	- Level	Difference^b^	Std err Dif	Lower CL	Upper CL	*p*-value^a^
Kinetic	Neural	131.226	42.704	19.979	242.474	0.0138
Exp	Neural	130.210	42.704	18.963	241.458	0.0148
Kinetic	OPLS	73.255	39.554	−29.786	176.296	0.2544
Exp	OPLS	72.239	39.554	−30.802	175.280	0.2661
OPLS	Neural	57.972	32.764	−27.383	143.326	0.2931
Kinetic	Exp	1.016	48.110	−124.315	126.347	1.0000

^a^ A *p*-value ≤ 0.05 considers the difference significant between the compared 2 groups, either model-model or model-experimental results.

^b^ This difference indicates the mean differences of the triplicate runs. On average, there are 108 different data points utilized to train the model, and validated with.

**TABLE 4:** Tukey’s HSD mean comparison of different model and experimental dataset for NSD.

	Level	- Level	Difference^b^	Std err Dif	Lower CL	Upper CL	*p*-value^a^
UDP-Gal	Kinetic	Neural	0.402	0.069	0.222	0.582	<.0001
	Exp	Neural	0.309	0.068	0.132	0.486	<.0001
	OPLS	Neural	0.241	0.065	0.072	0.409	0.0017
	Kinetic	OPLS	0.161	0.059	0.007	0.316	0.0366
	Kinetic	Exp	0.093	0.063	−0.070	0.256	0.4516
	Exp	OPLS	0.068	0.058	−0.082	0.219	0.6413
UDP-Glc	Neural	Kinetic	0.166	0.043	0.054	0.277	0.0010
	Neural	Exp	0.144	0.042	0.034	0.253	0.0046
	Neural	OPLS	0.140	0.040	0.036	0.245	0.0036
	OPLS	Kinetic	0.025	0.037	−0.071	0.121	0.903
	Exp	Kinetic	0.022	0.039	−0.079	0.123	0.9439
	OPLS	Exp	0.003	0.036	−0.090	0.097	0.9997
UDP-GalNAc	Neural	Kinetic	0.034	0.009	0.009	0.058	0.0027
	Neural	Exp	0.032	0.009	0.008	0.056	0.0035
	Neural	OPLS	0.029	0.008	0.008	0.051	0.0033
	OPLS	Kinetic	0.005	0.008	−0.017	0.026	0.9465
	OPLS	Exp	0.003	0.008	−0.018	0.024	0.9819
	Exp	Kinetic	0.002	0.009	−0.022	0.025	0.9983
UDP-GlcNAc	Kinetic	OPLS	0.129	0.019	0.085	0.173	<.0001
	Kinetic	Exp	0.097	0.023	0.043	0.151	0.0002
	Exp	OPLS	0.032	0.016	−0.008	0.071	0.1363

^a^ A *p*-value ≤ 0.05 considers the difference significant between the compared 2 groups, either model-model or model-experimental results.

^b^ This difference indicates the mean differences of the triplicate runs. On average, there are 108 different data points utilized to train the model, and validated with.

Multivariate data analysis (MVDA) with principal component analysis (PCA) on the data set was performed to ascertain the association of factors (extracellular metabolites) and responses (glycan and intracellular metabolites). A correlation matrix was made to analyze the relationship between the factors and the responses (Supplementary Figure SC4). For the MVDA analysis, SIMCA (Version 18.0, Sartorius, Cambridge, MA) was used.

## 4 Results

The results section is split into three categories (1) growth/production, (2) intracellular metabolite, and (3) glycan to understand the model predictive capability for each compartment.

### 4.1 Cell growth profile model prediction comparison

**FIGURE 3:**
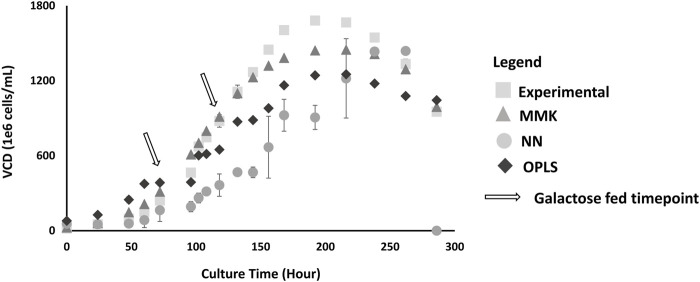
VCD Validation Plot; this plot depicts predictions by three different models: MMK (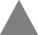), NN (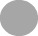), and OPLS (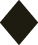) for comparison with experimental VCD (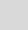). The experimental data is derived from the validation data set (n = 3) as shown in condition D in Table 1; with double galactose feed (25 mM) at 72 and 120 h. The standard error bars indicate the standard deviation simulated from the different model output or experimental dataset.

A comparative analysis between the three models was conducted. The results show that MMK provided the best fit for cellular growth and production (closely matching the experimental cellular growth parabolic trend, Figure 3). The NN model showed higher experimental deviation than MMK and OPLS models, displaying a more logarithmic (than parabolic) trend.

We also compared the mean difference with Tukey’s HSD (Table 2), the *p*-values indicate significant differences between the experimental validation and model output predictions. Interpreting the *p*-values is a bit counterintuitive as small *p*-values (≤0.05) are normally considered significant and favorable; however, in our comparisons (model output vs experimental values), this small *p*-value indicates unfavorable major deviations for model predictions. The Tukey’s HSD confirmed the observed graphical interpretation. MMK had a *p*-value close to 1 (0.9891), indicating an almost perfectly aligned prediction. Whilst the NN model showed the lowest *p*-value (0.0008) with OPLS also showing significant experimental differences; *p*-value (0.0650).

**FIGURE 4:**
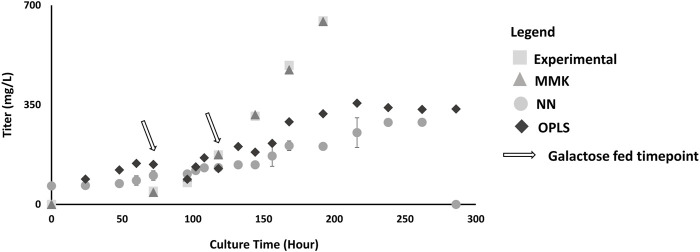
Titer Validation Plot; this plot depicts the model comparison between experimental validation (n = 3) as shown in condition D in Table 1; with double galactose feed (25 mM) at 72 and 120 h. The models are represented as: MMK (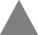), NN (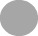), OPLS (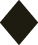) and experimental (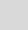) titer production. The standard error bars indicate the standard deviations simulated from the different model output (n = 3).

Furthermore, MMK best predicted mAb production (Figure 4). The NN and OPLS model resulted in unsatisfactory comparability after ∼120 h of culture time. The titer trend plateaus (∼330 mg/L) after 250 h for the NN model. Similarly, in titer prediction, the OPLS model observed several dips and recoveries (100 h, 120 h, 150 h), then dropped slightly after 200 h and plateaued (∼350 mg/L) around 250 h. Tukey’s HSD mean comparison (Table 3) also indicates significant differences observed with the NN compared to experimental (*p*-value of 0.0148). On the other hand, MMK displayed exceptional prediction (*p*-value = 1.0). OPLS model had moderate *p*-values of 0.25–0.29, indicating no significant differences.

MMK structure utilized glucose as the predominant variable in the cellular growth/production model structure. Which is a strong indicator of reliance of glucose for growth/production. We also examined for any correlation between variables with the NN profiler and OPLS correlation matrix to provide additional biological insights. The NN profiler (Supplementary Figure SC3) indicates glucose and ammonia are the main factors in predicting VCD and titer. The other remaining extracellular metabolites did not contribute to production and titer. Similar observations were seen in the correlation matrix (Supplementary Figure SC4); glucose correlates well with growth and production (−0.98 and −0.89, respectively). Only ammonia shows a positive correlation (0.75) with production and an insignificant impact on growth. In addition, the correlation matrix indicates a minor positive correlation (0.65) observed between glutamate and growth. Furthermore, the VIP plot (Supplementary Figure SC5) indicated a strong influence of glucose on the model prediction as well.

### 4.2 Intracellular metabolite model prediction comparison

**FIGURE 5:**
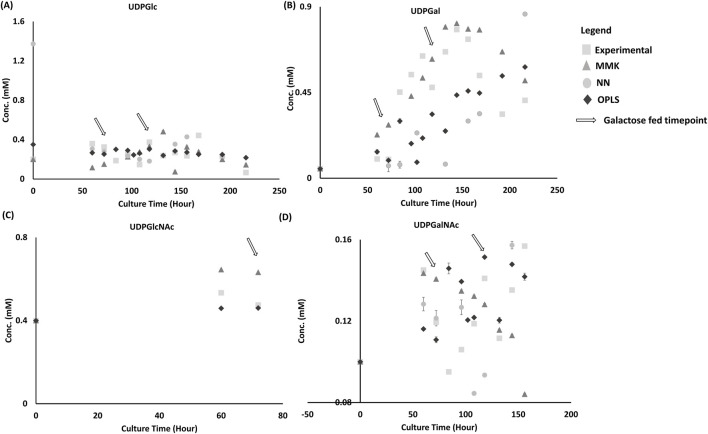
NSD Validation Set comparison between model and experimental dataset. From Figure 5, this plot depicts the prediction from three different models: MMK (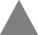), NN (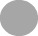), and OPLS (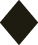) comparison from the validation experimental (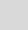) NSD set as shown in Table 1 as condition D; with double galactose feed (25 mM) at 72 and 120 h. Four intracellular metabolites were predicted: **(A)** UDP-Glc, **(B)** UDP-Gal, **(C)** UDP-GlcNCAc, **(D)** UDP-GalNAc with the experimental dataset. The standard bars indicate the standard deviation simulated from the different model output or experimental dataset (n = 3).^1^NN model was not representing UDP-GlcNAc due to the availability of the dataset, including it for prediction drove the NN model to skew the remaining validation data values (VCD, titer) to poor predication results (data not shown). ^2^GDP-Man and CMP-Sia were not used for model training and prediction because of its low abundance and reliability in data from HPLC analysis. ^3^The value at time 0 h for the neural network model prediction of UDP-Gal was not reported to simplify and aid in visualization of the model outputs and experimental data set with the available intracellular NSD, the predicted value is – 0.63755 mM (0 h), and −0.0635 mM (108 h).

The NN model prediction of intracellular nucleotide sugar donors (NSD) initial values was significantly off target for both UDP-Glc (>100%) and UDP-Gal (>100%), as shown Figures 5A, B, respectively. Moreover, UDP-Gal trend prediction captured the galactose feed increase at time 72 and 120 h, which is expected due to the utilization of galactose in the NN model and its positive correlation observed (Sha Sha and Yoon, 2019). However, the model could not predict the NSD consumption with UDP-Gal increasing to 0.9 mM (50% high) at ∼230 h. UDP-Glc (Figure 5A) model predictions fluctuate ± 0.4 mM from the steady experimental UDP-Glc of 0.2–0.4 mM. UDP-GalNAc had decent prediction trends, as shown in Figure 5D. OPLS had reasonable prediction trends observed for NSD (Figure 5). MMK had reasonable prediction trends observed for UDP-Glc and UDP-GlcNAc. But it had slight trouble predicting UDP-Gal and UDP-GalNAc dynamic trends.

Tukey’s HSD mean comparison (Table 4) reveals similar observations from the graphical interpretation for OPLS model having the least significance (UDP-Glc and UDP-GalNAc have *p*-values ≥0.94) amongst all the other models. UDP-Gal’s prediction was slightly worse for OPLS model (*p*-value 0.64). Whereas the OPLS model was not considered significantly different for UDP-GlcNAc (*p*-value of 0.1363). Unlike graphical observation, Tukey’s HSD observed to be insignificantly different for UDP-Gal and UDP-GalNAc (*p*-value of 0.45 and ≥0.94, respectively) for MMK. Likewise, reasonable graphical observations were statistically unfavorable, where UDP-GlcNAc significantly differed for the MMK model (*p*-value of 0.0002). In the case of the NN model, all NSD was considered significant (*p*-value < 0.005), with UDP-Glc being the least significantly different (*p*-value of 0.0046).

 From the correlation matrix (Supplementary Figure SC4), UDP-GlcNAc is the only observed NSD that can be correlated moderately well with extracellular metabolites (Glc (0.71), Glu (−0.76)). On the other hand, the NN profiler (Supplementary Figure SC3) describes the majority of the NSD having a correlation with Glc, Gln, Glu, Lac, and ammonia.

### 4.3 Glycan prediction model comparison

Overall, MMK significantly differed (*p*-values < 0.0001) for all glycan profile predictions. However, G2F was the least significant in glycan prediction among G0F and G1F (Figure 2). NN modeling adequately predicted G0F in the first half of the culture. However (>168 h), the model began to differ significantly (higher than anticipated G0F). The NN model predicted G1F well, with only one outlier at 144 h. NN model significantly differed for G2F (72 h (*p*-value = 0.039) and 144 h (*p*-value = 0.046)). OPLS model significantly differed for G0F for the first half of the process culture (<168 h). However, the prediction improved near the end of the culture. Likewise, similar observations can be seen for G1F (with predictions getting better ≥ 144 h). OPLS predicted G2F moderately well. The result we have observed between the three model predictions (growth and production, metabolites, and glycan profile) has some potential key differences between MMK, OPLS, and NN.

## 5 Discussion

This study developed and evaluated three different models: MMK, OPLS, and NN for prediction in 1) growth and production, 2) intracellular metabolites, and 3) glycan.

### 5.1 Cell growth & mAb production

The OPLS and NN models had similar titer predictions because of the different plateau distinctions at the later culture (>200 h). From Supplementary Figure C6, there is a correlative relationship between cell-specific productivity and specific glucose consumption during the growth phase. Others have observed a direct relationship between glucose impact on growth (Hsiao-Hsien et al., 2017). Hence, there was still an adequate prediction for the MMK model (Figures 3, 4), which was based solely on glucose (Supplementary Appendix BI). OPLS and NN model predictions were subpar, even though we trained the model with glucose data and even observed a high correlative relationship between glucose with VCD and titer (−0.98 and −0.89, respectively) from the correlation matrix (Supplementary Figure SC4). Furthermore, glucose was a dominant factor for the response prediction as indicated by the NN profiler (Supplementary Figure SC3). All three models indicate that glucose is a growth-limiting substrate (Bhagya and Sally, 2024). This indicates that certain aspects of the model with the given data (e.g., glucose) can be heavily weighted, and if constrained and defined properly, the data-driven or machine-learning models can provide more systematic realism like the kinetic model (Dongsheng et al., 2021).

The lack of prediction in the OPLS and NN model may also be due to missing data in the training set, 30% (VCD) and 75% (titer) (Supplementary Table SC1) (Ayilara et al., 2019; Emmanuel et al., 2021; Bayer et al., 2023). Unlike data-driven models, MMK was not limited by the missing data (Bayer et al., 2023). Excluding UDP-GlcNAc from NN resulted in better model prediction for VCD and titer (data is not shown). Hence, a lack of good data availability could result in lowered prediction of one or multiple response factors in a data-driven or machine-learning model (Apostolos and Ioscani, 2021; Bayer et al., 2023). Furthermore, the difference observed between the data-driven and kinetic models may have resulted from other metabolite inputs (e.g., ammonia) provided during the model training. In MMK, cellular growth/death did not account for the accumulation and toxicity produced by byproducts, e.g., ammonia or lactate, because it was unnecessary to include and would simplify the model structure. Although improvements can be made for cell lines that are sensitive to byproducts (e.g., lactate, ammonia, etc.) in the MMK model for cellular growth and death (Bhagya and Sally, 2024; López-Meza et al., 2016; Okamura et al., 2022). Furthermore, Selvarasu et al. and Yahia et al. have shown that certain AAs like arginine, threonine, serine, glycine, tyrosine, phenylalanine, methionine, histidine and asparagine, lysine, valine, and isoleucine were positively or negatively correlated with cell growth and mAb production (Ben Yahia et al., 2021; Selvarasu et al., 2010). Hence, it may be necessary for these data-driven and machine-learning models to include sufficient AA data sets for model training to develop better growth and production predictions. When coupled with rapid analytical tools, such as the REBEL for AA measurements (Chen et al., 2022), it can provide beneficial data for data-driven and neural network models with more rapid and readily available information. Harini et al. included specific uptake rates and showed improvement in the PLS model for growth and production predictions (Narayanan et al. (2019)). Clarke et al. have used -omics data, such as transcriptomics, to improve the prediction of growth and productivity for data-driven models (Colin et al., 2011).

### 5.2 Intracellular metabolites

Glutamine, glutamate, lactate, and glucose had more profound effects on the intracellular metabolites, according to the NN profiler (Supplementary Figure SC3). This observation could have been due to the glutamine and glutamate role in promoting the synthesis of macromolecules such as nucleotides (UTP, CTP); which is a building block for NSD (Song et al., 2006; Yoo et al., 2020). Furthermore, glucose is already a known feed that impacts NSD formation by forming intermediate substrates like glucose-6-phosphate (Sha Sha and Yoon, 2019; Yoo et al., 2020). Lactate is a byproduct during glucose’s conversion to glucose-6-phosphate. Furthermore, Liang et al. have demonstrated an indirect use of lactate feeding as the carbon source to manipulate certain target nucleotide sugar formation after glucose depletion (Liang et al., 2019).

Contradictory to what we had believed, the NN profiler (Supplementary Figure SC3) did not show a strong relationship between galactose and UDP-Gal (Figure 5B) (Sha Sha and Yoon, 2019). But from graphical observations, there was an observed positive change in UDP-Gal after the first feed (72 h) and a significant gradual increase in UDP-Gal due to the second galactose feed (120 h). Whereas the OPLS model predicted UDP-Gal much better and was more robust in capturing the system’s biological function (consumption and production) when feeding in additional galactose, portrayed as minor dips and increases (shown in Figure 5B) at ∼100 and 120 h. The correlation matrix showed a weak positive correlation between galactose (0.43) and UDP-Gal (Supplementary Figure SC4).

Furthermore, from Supplementary Table SC4, the % difference of the specific consumption of UDP-Gal_72-> 96 hr_ and UDP-Gal_118-> 144 hr_ indicates that the kinetic model portrays the best accuracy in terms of capturing the specific consumption/feed of UDP-Gal. This result coincides with Tukey’s HSD MMK model having one of the lowest deviations in both VCD and UDP-Gal predictions. According to Tukey’s HSD and graphical evaluation, NN model performance was the worst. However, when comparing the absolute values of the 1^st^ feed difference UDP-Gal_60-> 96 hr_ and the 2^nd^ feed difference UDP-Gal_96->144 hr,_ NN model’s average trend feed difference was the most accurate (32.65%), followed by OPLS (62.27%) and kinetic (103.62%). This observation can indicate that the machine learning models NN and OPLS follow the dynamic trend behavior of a system fed twice to be a lot more realistically than the kinetic model (e.g., UDP-Gal and UDP-GalNAc).

Although both OPLS and NN modeling are data-driven models, OPLS outperformed NN modeling in NSD predictions. This demonstrates that differences in model correlation for data-driven models (OPLS or neural) can result in different outcome predictions for NSD. Differences in the OPLS and NN models could be attributed to the approach. PLS uses the projection to latent space approach to model the linear covariance structure between the X and Y matrices (Liang et al., 2019). NN modeling consists of layered networks of interconnected mathematical operators (neurons). Here, each neuron acts as a weighted sum of the previous layer’s outputs transformed by an activation function (Liang et al., 2019). In addition, both models could be improved in their portrayal of the biological system by applying weights to the factor inputs or by standardizing the inputs (Liang et al., 2019; Rahul et al., 2022).

In addition, MMK trends for UDP-Gal were not simulated well when galactose was added during the experiment to modulate the UDP-Gal. Although a positive trend was observed, it is unclear whether it was due to the galactose addition or if the model captured multiple feedings of galactose, resulting in a continuous UDP-Gal increase (Figure 5). The kinetic model simulated the formation of intracellular NSD by utilizing intracellular glucose and glutamine values that were estimated from extracellular glucose and glutamine values transported to the cytoplasm based on parameter estimation. Extracellular glutamine and glucose had a clear indication of a correlation with intracellular NSD from both the OPLS matrix (Supplementary Figure SC4) and NN profiler (Supplementary Figure SC3). Hence, this lack of prediction trends could have been a result of the inappropriate kinetic model framework and the challenging parameter estimation required (Bayer et al., 2023).

### 5.3 Glycan

Overall, MMK differed significantly compared to the experimental (*p*-value < 0.0001); G2F had the least significant difference in terms of glycan prediction amongst G0F and G1F. The prediction performance was inadequate when compared to both the OPLS and NN models. Our result agrees with the previously demonstrated hybrid model; the kinetic model was outperformed by at least 30% in accuracy compared with a machine learning model for glycan prediction (Pavlos and Cleo, 2020). Machine learning models predict glycosylation profile changes more efficiently. This could have been attributed to the kinetic model structure complexity for parameter estimation and, consequently, a lack of nucleotide sugar prediction, resulting in an inaccurate or unemployable MMK prediction (Apostolos and Ioscani, 2021). However, the galactosylation index (Supplementary Figure SC7) at earlier time points (72 h and 96 h) had experimentally comparable trends. Thus, MMK’s capability to predict changes in galactosylation is still reliable. However, if strict control of the glycan profile is required, either a NN or an OPLS model would be more helpful.

Certain aspects of the OPLS model show a strong correlation between NSD and glycans (such as UDP-Gal with G0F (0.76), G1F (−0.77), and G2F (−0.7)). This relationship indicates that as UDP-Gal becomes a donor, the galactose is catalyzed by galactosyltransferase and provides the necessary galactose substrate for the maturation of precursor glycans (G0F) to form G1F and G2F (Gupta et al., 2023). In addition, certain extracellular metabolite(s) correlate well with those glycan forms, such as glucose (−0.84, 0.81, and 0.74, respectively). This could be the representation of the biosynthesis from glucose (*de novo* pathway) to GlcNAc-1P, in which the UDP-GlcNAc pyrophosphorylase catalyzes UTP and GlcNAc-1P to synthesize UDP-GlcNAc. The increase in UDP-GlcNAc availability can provide additional substrates for G0F formation by the catalyzation of GlcNAc by N-acetylglucosaminyltransferases (Fan et al., 2015; Khoder-Agha et al., 2019; Qiao et al., 2021). The accumulated abundance of UDP-GlcNAc was always equal to or above the levels of both UDP-Glc and UDP-Gal (Supplementary Figure SC8).

Furthermore, OPLS and NN models can circumvent the need to obtain NSD concentrations to sufficiently predict glycan profiles. Indirectly, OPLS and the NN model utilized just extracellular metabolites to predict glycan profiles because of their indirect relationship with certain NSD, such as glucose and glutamate, as observed in the correlation matrix (Supplementary Figure SC4) and by others (Ben Yahia et al., 2021; Selvarasu et al., 2010; Green and Glassey, 2015). Glucose has a correlation value of 0.71 for UDP-GlcNAc and −0.62 for UDP-Gal. Glutamate had a correlation of −0.76 for UDP-GlcNAc. Some correlation can be seen with galactose for UDP-Gal (0.43) and UDP-GalNAc (0.36). Due to the lack of data (Reference Supplementary Table SC1), the corresponding NSD correlations with galactose could be impacted (János et al., 2021; van Ginkel et al., 2023).

In addition, the decrease in G1F and G2F observed with higher glucose concentrations can be due to the rate limitation of galactose consumption. Different sugars have been observed to have different transport rates, especially when multiple sugars are prevalent in the media (e.g., a mixture of glucose and mannose feed would result in a qs_glc_ > qs_man_) (Zhang et al., 2021). Hence, early in the culture (<120 h), the abundance of glucose may have impacted the transport rate of galactose. From the raw validation dataset (Sha Sha and Yoon, 2019), with the initial galactose fed at 72 h (culture period 72 h -> 120 h), the qs_glc, 1 (72-> 120 h)_ = −5.703 × 10E-5 mM/(cell*h), the qs_gal, 1 (72 -> 120 h)_ = −1.708 × 10E-5 mM/(cell*h). The second galactose fed was conducted at 120 h (culture period 120 h -> 170 h), the qs_glc, 2 (120 -> 170 h)_ = −4.384 × 10E-5 mM/(cell*h), and the qs_gal, 2 (120 -> 170 h)_ = −2.700 × 10E-5 mM/(cell*h). There was a 1.5x fold decrease in qs_glc, 1 -> 2_, a 1.5x fold increase for qs_gal, 1 -> 2,_ and a 2x decrease in glucose abundance. This finding suggests that there is an impact on the galactose uptake rate and, subsequently, galactosylation due to the prevalence of glucose.

From the NN profiler (SSupplementary Figure SC3), galactose did not majorly impact glycan forms. However, it is expected that galactose should have a major contribution to forming necessary glycan precursor metabolites (e.g., UDP-Gal) (Sha Sha and Yoon, 2019), which will play a key role in galactosylation. From our dataset, we have observed some correlation with galactose (Supplementary Figure SC4). Thus, it is surprising that no correlation was observed between galactose and glycan profile (in which even glucose had some correlations). Hence, this indicates that there may be some limitations to using extracellular metabolites to predict glycan profiles. Unlike glucose measurements, labs are more predisposed to have a rapid at-line analytical tool to assess its concentration (e.g., Nova Flex or YSI instruments). On the other hand, galactose measurements have not been fully implemented. Hence, a lack of good available datasets could result in less correlative relationships and its use for prediction, model training and extrapolation (János et al., 2021). Recently, CEDEX Bio has created an at-line instrument that simultaneously allows galactose and glucose abundance detection (Zhang et al., 2021).

These findings support the correlative relationship between extracellular metabolite and glycan, and the possibility of data-driven/neural models to extrapolate and provide some additional interpretation for the biological system. Furthermore, extracellular metabolites can be utilized indirectly to predict glycan profiles and are more beneficial because of their prominent availability compared to intracellular metabolite measurements.

In addition, intracellular metabolites are limited by their detection accuracy and sample processing, where differences in extraction and quantification of the metabolites can relay different results in measurements (Andresen et al., 2022). Included in our training dataset (Supplementary Table SC1), measurements for intracellular NSD were not as robust as extracellular metabolites (87% missing UDP-GlcNAc data, while other NSD have 55% of missing data, and extracellular metabolites have 30%–40% missing data). Data-driven models can be skewed or misinterpreted due to the current analytical tools to measure intracellular metabolites (as we have observed for UDP-GlcNAc on the NN model).

Although part of the model results are considered significant, clinical evaluation must be factored into these models to understand the relevance. Clinical data and evaluation of different mAbs from different batches have shown drastic changes in the glycosylation profile and have demonstrated not to have an impact on its clinical efficacy and safety (Xie et al., 2010; Schiestl et al., 2011; Beck et al., 2012; Batra and Rathore, 2016). Different batches of Enbrel ® (pre-production and post-production expiration) found that G2F decreased from ∼50% to ∼30%, respectively. Theoretical ADCC activity would have been impacted by a factor of ∼1.5 (Schiestl et al., 2011; Thomann et al., 2016). However, these drugs were not recalled back from the market, indicating no clinical difference observed with 20% change in galactosylation. Rituxan/MabThera, innovator drugs, and biosimilar drugs displayed similar observations (Xie et al., 2010; Schiestl et al., 2011). Hence, the variation observed in MMK, OPLS, and NN models at the earlier time points may be negligible and insignificant even though the prediction accuracy is considered significant. Furthermore, the calculated galactosylation index from each model prediction (Supplementary Figure SC7) is <20% compared to the experimental, which could be insignificant from a clinical efficacy and safety standpoint.

Therefore, these models are best used to understand trends and implications affected by process or feed changes. Some limitations to the kinetic based model would require fine tuning and optimization for cell line specific models (e.g., framework). It would be more advantageous to utilize more rapid methods like OPLS or NN models to predict and understand how a factor can manipulate the glycan profile. However, these data-driven models require the necessary data (e.g., extracellular metabolites: galactose, glycan: HM, etc., process parameters (DO, pH), enzymes, and cofactors (metals)) and to minimize missing data and screen out arbitrary data (e.g., UDP-GlcNAc in some cases). In addition, all models would require clinical data to provide realistic, and meaningful interpretation. This may also limit the use of a sole kinetic model in its performance to capture clinical impact factors.

## 6 Conclusion

This study compares soft-sensor modeling used for biopharmaceutical CQA monitoring (summarized in Table 5), revealing high-accuracy MMK predictions for growth and production with moderate intracellular metabolite accuracy. Diminished measurement precision is seen when predicting glycan profile absolute values, demonstrating moderate success in trend prediction. In contrast, the OPLS and NN models show higher capabilities in glycan profile predictions despite their limitations in estimating growth and mAb production. Notably, the kinetic model relies solely on glucose for its calculations, whereas the OPLS and NN models may necessitate additional metabolite inputs, such as amino acids and specific consumption rates, for improved accuracy.

**TABLE 5:** Summary for the different models results^a^.

Model type	Growth/Production	Intracellular metabolites (NSD)	Glycan
Kinetic (MMK)	Satisfactory trend predictions for both growth and production. In addition, the simulated results showed high accuracy (deviations <5%). Requires minimal data inputs (e.g., only glucose)	Trend predictions were not decent and had trouble with predicting polynomial datasets (e.g., UDP-Gal/UDP-GalNAc). Dependent on the dataset, accuracy varies with the pattern of the experimental data sets (deviations 5%–50%)	Early trend predictions (72, 96 h) for G0F, G1F were satisfactory. However, deviations on the trend predictions were not satisfactory <96 h. The accuracy (>96 h predictions) was unsatisfactory (20%–90%)
OPLS	Satisfactory trend predictions for growth and moderate trends predictions for production. However, it was lacking in accuracy for titer predictions. Growth had adequate prediction accuracy (deviations >5%). Requires extensive data inputs for reasonable predictions (e.g., AA, glucose)	Satisfactory trend predictions for each NSD. However, slight prediction of the UDP-Gal could be improved, where the model tends to accumulate after the feed, and the model had trouble considering the consumption of NSD. There was good accuracy in NSD predictions (deviations 5%–10%)	Satisfactory trend predictions, and accuracy (deviations 5%–10%). Initial trouble with prediction in the early time points 72, 96 h due to a lack of initial constraint in data
Neural (NN)^b^	Lacked in both trend and accuracy (deviations <50%) predictions for growth and productions. The simulated output variables have higher standard deviations at individual data points. Requires extensive data inputs for reasonable predictions (e.g., AA, glucose)	Results varied amongst the different NSD. Satisfactory simulation trends for NSD to simulate galactose supplementation. However, higher deviations were observed after 250 h. In addition, the model lacks in accuracy for UDP-Gal predictions (deviations >50%). Not capable of simulating output variables that have >87% missing variables (UDP-GlcNAc)	Same predictability as OPLS. However, higher variability in data predictions were observed at individual time points

^a^ Trends follow the experimental result shapes (e.g., linear, polynomial, etc.). Accuracy is the indication of the difference in absolute values between the experimental and model output results.

^b^ Model misses certain predicted output results (e.g., VCD_286_, Titer_286,_ UDP-Gal_84, 102, 132, 168_, etc.).

The quality of its training dataset significantly influences the NN model’s predictive strength, particularly for intracellular NSD. On the other hand, MMK and OPLS models demonstrate a degree of robustness, are less affected by data quality and achieve reliable predictions for intracellular NSD. By facilitating the prediction of intracellular NSD, data-driven models enable extrapolation and indirect biological system insights, revealing connections among nucleotide sugars, glycans, and the synthesis of extracellular metabolites like glutamine and glucose. This capability indicates that, given appropriate datasets, data-driven models can shed light on complex biological systems and assist in extrapolating new processes, such as the effects of double galactose addition on NSD and glycosylation. Therefore, our study shows that data-driven models have the potential to predict beyond their training scope, offering valuable insights into biological systems that could inform process optimization. This is facilitated by tools like JMP’s (NN) profiler or the OPLS correlation matrix. We anticipate that data-driven approaches, including OPLS and machine learning models, will increasingly surpass kinetic models in biological functionality prediction and extrapolation. This advancement is expected as data sharing grows, integrating diverse datasets from -omics, clinical relevance, rapid metabolite measurements, and extracellular metabolites. Moreover, the biological system insights garnered from these models can enhance kinetic-based models, highlighting the complementary roles of different modeling approaches in advancing bioprocess understanding and optimization.

## Data Availability

The original contributions presented in the study are included in the article/Supplementary Material, further inquiries can be directed to the corresponding author.
