# Supplementary material for: Soft-sensor model development for CHO growth/production, intracellular metabolite, and glycan predictions
Source: Front Mol Biosci. 2024 Oct 22;11:1441885. doi: 10.3389/fmolb.2024.1441885 (PMC11535473; doi:10.3389/fmolb.2024.1441885)
Supplement: Supplementary file 1 [file DataSheet1.pdf]

**Appendix A. Design of experiment Supplementation**

Table 1 conditions are previously published in the following manuscript from Sha et al. [2]. 0 or 25 mM galactose and single versus double feed conditions were chosen based on an indepdent study published in the same manuscirpt [2]. In brief, an independent design of experiment (DoE) that constisted of 0, 20, and 40 mM galactose were fed at 72 hours. There were promising results in terms of cellular growth and production (mainly due to the extended cell culture) from galactose fed conditions; with 40 mM galactose performance being the best. Hence, a later DoE was conducted to investigate both time and additional galactose feed's impact on glycosylation and its dyanmics with nucleotide sugar donors (as an important intermediate substrate for galactosylation upregulation). This design, which is detailed in Table 1, consists of investigating single feeds (total 25 mM galactose) at either 72 or 120 hours or with a double feed by feeding at both 72 and 120 hours (total 50 mM galactose).

**Appendix B. Model Development Supplementary Documentation**

I. **Kinetic Model (MMK) development:**

The modified model is based on the reference model (Jedrzejewski, et al. 2014) [14]. The modifications made herein were specific for our cell line and supplementation strategy and data availability.

a) **Growth & Death Kinetics:**

Growth μ ($hr^{-1}$) and death kinetics $k_d$ ($hr^{-1}$) were based on Monod kinetics that relates to maximum growth $\mu_{max}$ ($hr^{-1}$) and death $kd_{max,Glc}$ ($hr^{-1}$), glucose extracellular concentration $Glc_{Ext}$ (mM) and a Monod constant Km (mM).

$$\mu = (\mu_{max})\left(\frac{[Glc_{Ext}]}{(Km_{x,Glc} + [Glc_{Ext}])}\right) (1)$$

$$k_d = (kd_{max,Glc})\left(\frac{Kdd_{Glc}}{(Kdd_{Glc} + [Glc_{Ext}])}\right) \quad (2)$$

From OPLS analysis (Figure C4 & C6), glucose was used in the model as the sole contributor for growth and titer production as it had a direct negative correlation for viable cell density ($X_v$) and titer production (mAb). $X_v$ (cells·$mL^{-1}$) profile of cell is modeled as the following:

$$\frac{d(VX_v)}{dt} = (\mu - k_d)\, X_v\, V - F_{out}X_v \quad (3)$$

With consideration of the feed F (mL·hr$^{-1}$), volume V (mL) for sampling or addition into the reactor. The productivity mAb (mg·L$^{-1}$) of the protein production is a function of its yield $q_{prod}$ (mg·L$^{-1}$).

$$\frac{d(V[mAb])}{dt} = Y_{prod,NG} X_v V - F_{out}[mAb] \quad (4)$$

Extracellular metabolites were modeled based on a feed in flow rate $F_{in,feed}$ (hr$^{-1}$) per glucose concentration going in $Glc_{conc,in}$ (mM), taken in account of the specific consumption rate Q (mmol· (h-cell)$^{-1}$) required for cell growth and flow rate out $F_{out}$ (hr$^{-1}$) of the current extracellular glucose concentration $Glc_{Ext}$ (mM).

$$\frac{d\,(V\,[Glc_{Ext}])}{dt} = F_{in,feed}\, Glc_{conc,in} - Q_{Glc}\, X_v V - F_{out}\,[Glc_{Ext}] \quad (5)$$

The specific consumption rate is a function of growth per yield of coefficient required for growth $Y_{x,Glc}$ (cell·mmol$^{-1}$), in addition to a maintenance constant m (mmol· (h-cell)$^{-1}$) that is required for other essential cell metabolism and functions such as generating energy, Kreb cycle etc.

$$Q_{Glc} = \frac{\mu}{Y_{x,Glc}} + m_{Glc} \quad (6)$$

However, the specific consumption of galactose was modified to account for conditions of different feeding timepoints strategy in our model. Here, when there is no galactose fed into the cell culture, cell growth and death is neither affected by the specific rate of galactose consumption. This is dictated by the following equation ($\frac{Gal_{Ext}}{(Gal_{Ext}+Km_{x,Gal})}$) that involves the concentration of galactose $Gal_{Ext}$ (mM) and its Monod kinetic constant $Km_{x,Gal}$ (mM).

$$Q_{Gal} = \left(\frac{\mu}{Y_{x,Gal}} + m_{Glc}\right) * \frac{Gal_{Ext}}{(Gal_{Ext} + Km_{x,Gal})} \quad (7)$$

As $Gal_{Ext}$ << 0, the death and the growth of the cells is minimal, which depicts the earlier cell growth of the cells with an initial feed of the galactose. Likewise, as the cells consume more galactose, the cells are at a later stage of the production, which results in lower growth and death, as it follows the same trend as seen in the experimental datasets from CHO-K1. Whereas, when Cgal ~= 0, the rate of the cell death and growth is not affected by parameters that involves galactose.

**b) <u>Intracellular metabolite network:</u>**

Simplified KEGG Pathway maps were used to mimic the intracellular metabolite networks with molecular interaction and reactions. To reduce computational time and improve model accuracy, only known

quantifiable metabolites (mainly nucleotide sugars) were used in the model framework. Nonetheless, intermediate metabolites such as Glucose-6-P, Fructose-6-P and GDP-4 keto-D-Rha were kept in the model framework because of its reversible metabolism reaction pathway with key nucleotide sugar metabolites. Intracellular metabolites were represented as the rate of specific consumption per cell volume $V_{Cell}$ (mL · cell$^{-1}$))) taken into each cell and subtracted from the nucleotide sugar formation reaction rates r (mM · hr$^{-1}$) required for nucleotide sugar donor formation.

$$\mathrm{Glc_{Int}} = \frac{\mathrm{Q_{Glc}}}{\mathrm{V_{cell}}} - \left(\mathrm{N_{Glc\_ATP}} * \mathrm{r_{1aF}} + \mathrm{N_{Glc_{GDPMan}}} * \mathrm{r_{4aF}} + \mathrm{r_{met\_gal}}\right) \quad (8)$$

$$\mathrm{r_{met\_glc}} = \frac{\mathrm{Q_{Glc}}}{\mathrm{V_{cell}}} * \mathrm{f_{Glc}} \quad (9)$$

Whereas the nucleotide sugar formation reaction rates are depicted as Michaelis Menten reactions; with the rate of enzyme turnover k (hr$^{-1}$), enzyme initial concentration $E_0$ (mM), nucleotides concentration (ATP, UTP, etc.) (mM), Michaelis Menten constants Km (mM).

$$\mathrm{r_{1aF}} = (\mathrm{k_{1aF}} * \mathrm{E_0} * ATP) * \left(\frac{\mathrm{Glc_{Int}}}{(\mathrm{KM_{1aF_{ATP}}} * \mathrm{KM_{1aF_{glcext}}} + \mathrm{KM_{1aF_{glcext}}} * \mathrm{ATP} + \mathrm{ATP} * \mathrm{Glc_{Int}})}\right) \quad (10)$$

### c) <u>**Golgi maturation network:**</u>

The kinetic structure of the glycosylation was derived based on the N-linked glycosylation model for hybridoma cell line from Jedrzejewski et al. [14]. The hybridoma model included different types of kinetic equations: single substrate uni-uni enzyme kinetics, random order bi-bi enzyme kinetics, ordered bi-bi enzyme kinetics, ping-pong bi-bi enzyme kinetics, and ping-pong ter-ter enzyme kinetics. Therefore, to simplify the model, only high mannose glycan production was based on single substrate uni-uni enzyme kinetics, because the progression of glycan maturation was solely formed from the precursor glycan. An example such as when one mannose is enzymatically cleaved with mannosidase I from high mannose 9 (Man9) to form high mannose 8 (Man8), where the enzyme concentration is represented as E0 (mM) and the enzyme turnover rate k (hr-1). With Michaelis Menten kinetic constant KM (mM), and the high mannose 9 precursor substrate as cA (mM).

$$r_{(Man9 \to Man8)} = \frac{E_0 * k_{cat} * c_A}{(K_M + c_A)} \quad (11)$$

Whereas the remaining glycan maturation is defined as a Bi-ternary complex Michaelis Menten kinetic reaction $r$, which assumes the enzymatic reactions $c_{enz}$ (mM) that occur with two substrates $c_{A/B}$ (mM) pertaining to either the nucleotide sugar donor or the precursor glycan. It is either an ordered or random sequential mechanism and has an enzyme turnover rate of $k$ ($hr^{-1}$). With Michaelis Menten kinetic constants as $K_{AM/BM}$ (mM) and inhibitory kinetic constants $K_{AI}$ (mM).

$$r = \frac{c_{enz} * k_{cat} * c_A * c_B}{(K_{AI}K_{BM}) + (c_A * (K_{\boldsymbol{BM}}) + (K_{AM} * c_B) + (c_A * c_B)} \quad (12)$$

The transport of nucleotide sugar donor was assumed to have a constant fluxed transport from the nucleus to the Golgi. Byproduct formation was neglected to simplify the model computation time and assumed to be negligible. Thirty-five glycan structures are simulated starting from Man9 to more complex sialylated N-glycan forms (G2FS2) by a cascade of kinetic equations. Initial glycans were assumed to start from Man9 that is defined as the specific productivity $q_{prod}$ (mg · cell-1), the molecular weight of the monoclonal antibody MW (mg · mmol-1), and volume of the cell Vcell (L · cell-1). The MW was based as 150,000 mg · mmol-1. Since glycosylation typically occurs on the two heavy chains, a factor of 2 was considered into the equation.

## II. <u>Neural Network (NN) Model JMP Code:</u>

```
/* Neural SAS Scoring*/
/*%PRODUCER: JMP - Neural */
/*%TARGET: G0F */
/*%TARGET: G1F */
/*%TARGET: G2F */
/*%TARGET: VCD */
/*%TARGET: UDP-Gal */
/*%TARGET: UDP-Galnac */
/*%TARGET: UDP-Glc */
/*%INPUT: Amm */
/*%INPUT: Glc */
/*%INPUT: Gln */
/*%INPUT: Glu */
/*%INPUT: Lac */
/*%INPUT: Gal */
/*%OUTPUT: G0F_Predicted */
/*%OUTPUT: G1F_Predicted */
/*%OUTPUT: G2F_Predicted */
/*%OUTPUT: VCD_Predicted */
/*%OUTPUT: UDP-Gal_Predicted */
/*%OUTPUT: UDP-Galnac_Predicted */
/*%OUTPUT: UDP-Glc_Predicted */
LABEL G0F_Predicted = 'Predicted: G0F';
LABEL G1F_Predicted = 'Predicted: G1F';
LABEL G2F_Predicted = 'Predicted: G2F';
LABEL VCD_Predicted = 'Predicted: VCD';
```

```
LABEL UDP-Gal_Predicted = 'Predicted: UDP-Gal';
LABEL UDP-Galnac_Predicted = 'Predicted: UDP-Galnac';
LABEL UDP-Glc_Predicted = 'Predicted: UDP-Glc';
/* Transformation Code */
/* Hidden Layer Code */
H1 = tanh(.5*(0.144435388867055*Amm + -0.099697385924605*Glc + -
0.0729060877525817*Gln + 0.0744423359569146*Glu + -0.0647248820855939*Lac + -
0.00948660618288411*Gal + 0.767867813459082));
H2 = tanh(.5*(-0.0241279855637704*Amm + 0.102376244934949*Glc +
0.0244001074394888*Gln + -0.091273167300053*Glu + 0.0705854291330278*Lac +
0.00935727111085235*Gal + -0.959451453215597));
H3 = tanh(.5*(0.114503045662866*Amm + 0.0919883417160336*Glc + -
0.0349595470421446*Gln + -0.111563046261703*Glu + 0.0851720368859003*Lac +
0.00795857537326395*Gal + -0.887891367754835));

/* Final Layer Code */
THETA1=-3.13371572033752*H1 + -6.88265685312173*H2 + 3.9690639373779*H3 +
0.751719031850651;
THETA2=2.57879617432819*H1 + 5.7358573470468*H2 + -3.33541201175486*H3 +
0.213286735723026;
THETA3=1.08442476263036*H1 + 2.14323311030728*H2 + -1.16403279506524*H3 +
0.0518257063653169;
THETA4=56068.6934239867*H1 + -31864.8605488242*H2 + -36560.6579205286*H3 +
102505.129342009;
THETA5=18.9780803874085*H1 + 28.4231896340067*H2 + -12.4045265993519*H3 +
1.76330229844312;
THETA6=-3.40596262220832*H1 + -6.01149913307488*H2 + 3.04763345929546*H3 + -
0.0210699160691764;
THETA7=-15.543843144693*H1 + -25.8474777201843*H2 + 12.4397425212281*H3 + -
0.633668442203987;

/* Response Mapping Code */
G0F_Predicted = THETA1;
G1F_Predicted = THETA2;
G2F_Predicted = THETA3;
VCD_Predicted = THETA4;
UDP-Gal_Predicted = THETA5;
UDP-Galnac_Predicted = THETA6;
UDP-Glc_Predicted = THETA7;
```

**Appendix C. Appendix Figures & Tables**

**Figure C1**. Summary of model fit for the OPLS model based on NSD training dataset availability.

This figure C1, indicates the cumulative (R2X & Y) and cumulative predictive (Q2) model. A) The model has a R2X (cum) of 0.954 and a R2Y (cum) of 0.75 and a Q2 (cum) of 0.684. This model utilizes NSD as a component for glycan prediction. B) This model did not utilize NSD as a component for glycan prediction, but as a response variable. The model has a R2X (cum) of 0.973 and a R2Y (cum) of 0.591 and a Q2 (cum) of 0.34. Five components were utilized for this OPLS model. Subsequent analysis with ANOVA was conducted with OPLS

model B because it gave better prediction for the response variables. The training of the OPLS model used 3 sets of training based on Table 1 (control - no feed, condition A – galactose feed at 72 hours and B – galactose feed at 120 hours). The model was then validated with a condition C – double galactose feed at 72 and 120 hours.

**Figure C2**. OPLS validation prediction trends of glycan dependent on NSD training dataset availability. Experimental (■), and OPLS (◆) representation for glycan prediction comparison based on the validation set; condition D in Table 1. **(A)** model utilized NSD as a training dataset for glycan prediction, **(B)** model did not utilize NSD as a training dataset for glycan prediction.

From Figure C2, the validation set (D) from Table 1 was used for model validation. Model B had better glycan prediction trends for extrapolation of the data. Where G0F tends to increase over the culture course without any additional feeding, G1F and G2F should be decreasing.

**Figure C3.** Neural network (NN) model Profiler

Figure C3 shows the NN model's correlation between the input variables (extracellular metabolites) with the model outputs (growth, production, NSD, and glycans). A flat line indicates no relationship can be seen at different concentrations for the inputs to outputs. Indirectly, in this plot, single input coordinates can be used to inform and ascertain certain output scenarios (e.g., 0.6 mM Lac at 20 g/L glucose would result in 0.04% G2F).

**Figure C4.** Correlation Matrix between metabolites, process variables, and quality attributes

Figure C4 is a correlation matrix between extracellular metabolites, intracellular metabolites and measured product quality attributes determined by multivariate data analysis. The cells with thick boundary lines show interesting observations between process parameters and the quality attributes of the mAb. Correlations between intracellular metabolites (UDP-Gal, UDP-GalNAc) and extracellular metabolites (Glc) and glycan profiles can be observed. Similar observations can be seen for titer and VCD with Glc.

**Figure C5.** VIP contribution from OPLS model based on NSD training dataset availability.

From Figure C5, the variable of importance plot (VIP) indicates which factor variables are the most important for response variable predictions. The VIP plot is generated from the same model output from Figure C1. A)

represents the OPLS model that considers NSD as a factor variable, B) represents the OPLS model that considers NSD as a response variable. By comparing the VIP between the two models, glucose is used for both glycan and NSD prediction (high correlation). Glutamate and glutamine are considered as important factor variables for both models as well. Looking at model A, UDP-Gal and UDP-Glc, UDP-GalNAc are more important factors to consider for glycan prediction. The low VIP value for UDP-GlcNAc may have been a result of missing data that resulted in low correlation or importance in this NSD variable.

**Figure C6**. Cell specific productivity versus specific glucose consumption

From Figure C6, the specific glucose and cell specific productivity was analyzed during the exponential growth phase from period from 96 hours to 168 hours. A $R^2$ of 0.9701 was observed between the specific glucose uptake rate and cell specific productivity.

**Figure C7.** Time Series Galactosylated index comparison between models[1,2]

From Figure C7, this plot depicts 3 different models: with one experimental ( ), MMK ( ), NN ( ), and OPLS ( ) for galactosylation index on the validation model prediction set; as shown in Table 1 as condition D (25 mM galactose fed at 72 and 120 hours).

[1]NN model galactosylated index is missing at 168 hours, because there was no prediction for G0F and G2F. Including just prediction values for G1F, provided unreasonable index results.

[2]The galactosylated index was calculated based on the following equation: $\text{Galactosylation index (GI)} = \frac{(0*\,\text{G0F}+1*\,\text{G1F}+2*\text{G2F})}{2*(\text{G0F}+\text{G1F}+\text{G2F})}$

From Figure C7, the overall trends observed for the models capture the experimental trend. The absolute values of the galactosylated index were predicted well for NN, OPLS and MMK; except for the kinetic model significantly differing >150 hours.

**Figure C8**. Training Dataset model prediction comparison for different models

Figure C8, the training Dataset comparison based on the feeding conditions on Table 1 for extracellular/intracellular metabolites, growth/production and glycan predictions, A) Control (no feed); B) Feed A with single galactose supplement at 72 hours; C) Feed B with single galactose supplement at 120 hours. For each condition A-C, each of the models are represented as the following: experimental ( ), MMK (

▲ ), NN (●), and OPLS (◆). NN and OPLS models do not have training data sets for extracellular metabolites because the software tools did not have the capability to physically predict input values (unlike kinetic model framework). In addition, data represented for NN and OPLS model predictions does not include NSD as an input.

**Appendix Tables**

**Table C1**. Reference for the data miscellaneous missing from SIMCA

| | N | Missing (%) | Mean | Std. dev. |
|---|---|---|---|---|
| **Amm** | 75 | 32.4324 | 1.454 | 1.09833 |
| **Glc** | 64 | 42.3423 | 20.599 | 11.0491 |
| **Gln** | 75 | 32.4324 | 5.26493 | 1.13078 |
| **Glu** | 75 | 32.4324 | 4.0768 | 0.60622 |
| **Lac** | 70 | 36.9369 | 0.332429 | 0.483713 |
| **Gal** | 57 | 48.6487 | 3.046 | 7.43866 |
| **G0F** | 26 | 76.5766 | 0.735975 | 0.103004 |
| **G1F** | 26 | 76.5766 | 0.233272 | 0.0851673 |
| **G2F** | 22 | 80.1802 | 0.0363436 | 0.0166498 |
| **UDP-Gal** | 50 | 54.955 | 0.166079 | 0.169628 |
| **UDP-GalNAc** | 50 | 54.955 | 0.117032 | 0.0238028 |
| **UDP-Glc** | 50 | 54.955 | 0.25904 | 0.0521156 |
| **UDP-GlcNAc** | 14 | 87.3874 | 0.455511 | 0.0399504 |
| **VCD** | 78 | 29.7297 | 66523.7 | 55771.1 |
| **Titer** | 28 | 74.7748 | 184.742 | 171.694 |

Table C1 indicates the total number of datapoints (N) provided for each variable. Each variable has a missing value defined as a percentage based on the total number of datapoint from all the training sets (108 datapoints). The mean is the average of all the training sets' datapoints. Standard deviation is defined from all the training sets' datapoints.

**Table C2**. Output Variable Training and Validation R2 and Log likelihood for Neural network (NN) model

| **Output Variable** | **Training** | | **Validation** | |
|---|---|---|---|---|
| **G0F** | Measures | Value | Measures | Value |
| | RSquare | 0.924 | RSquare | 0.929 |

| | | | | |
|---|---|---|---|---|
| | RASE | 0.025 | RASE | 0.009 |
| | Mean Abs Dev | 0.019 | Mean Abs Dev | 0.009 |
| | -LogLikelihood | -25.090 | -LogLikelihood | -9.753 |
| | SSE | 0.007 | SSE | 0.000 |
| | Sum Freq | 11.000 | Sum Freq | 3.000 |
| **G1F** | Measures | Value | Measures | Value |
| | RSquare | 0.925 | RSquare | 0.825 |
| | RASE | 0.021 | RASE | 0.014 |
| | Mean Abs Dev | 0.018 | Mean Abs Dev | 0.012 |
| | -LogLikelihood | -26.998 | -LogLikelihood | -8.516 |
| | SSE | 0.005 | SSE | 0.001 |
| | Sum Freq | 11.000 | Sum Freq | 3.000 |
| **G2F** | Measures | Value | Measures | Value |
| | RSquare | 0.810 | RSquare | 0.552 |
| | RASE | 0.007 | RASE | 0.006 |
| | Mean Abs Dev | 0.006 | Mean Abs Dev | 0.004 |
| | -LogLikelihood | -39.415 | -LogLikelihood | -10.953 |
| | SSE | 0.000 | SSE | 0.000 |
| | Sum Freq | 11.000 | Sum Freq | 3.000 |
| **VCD** | Measures | Value | Measures | Value |
| | RSquare | 0.989 | RSquare | 0.976 |
| | RASE | 5517.676 | RASE | 2782.727 |
| | Mean Abs Dev | 4069.434 | Mean Abs Dev | 2057.087 |
| | -LogLikelihood | 421.455 | -LogLikelihood | 46.751 |
| | SSE | 1280000000.000 | SSE | 38717849.000 |
| | Sum Freq | 42.000 | Sum Freq | 5.000 |
| **Titer** | Measures | Value | Measures | Value |
| | RSquare | 0.693 | RSquare | . |
| | RASE | 61.136 | RASE | 84.581 |
| | Mean Abs Dev | 56.756 | Mean Abs Dev | 84.581 |
| | -LogLikelihood | 66.384 | -LogLikelihood | 5.857 |
| | SSE | 44850.917 | SSE | 7153.916 |
| | Sum Freq | 12.000 | Sum Freq | 1.000 |
| **UDP-Gal** | Measures | Value | Measures | Value |
| | RSquare | 0.669 | RSquare | . |
| | RASE | 0.113 | RASE | 0.078 |
| | Mean Abs Dev | 0.092 | Mean Abs Dev | 0.057 |
| | -LogLikelihood | -18.363 | -LogLikelihood | -5.641 |
| | SSE | 0.304 | SSE | 0.031 |

| | | | | |
|---|---|---|---|---|
| | Sum Freq | 24.000 | Sum Freq | 5.000 |
| UDP-GalNAc | Measures | Value | Measures | Value |
| | RSquare | 0.683 | RSquare | . |
| | RASE | 0.012 | RASE | 0.008 |
| | Mean Abs Dev | 0.010 | Mean Abs Dev | 0.006 |
| | -LogLikelihood | -74.690 | -LogLikelihood | -16.832 |
| | SSE | 0.004 | SSE | 0.000 |
| | Sum Freq | 25.000 | Sum Freq | 5.000 |
| UDP-Glc | Measures | Value | Measures | Value |
| | RSquare | 0.671 | RSquare | . |
| | RASE | 0.028 | RASE | 0.040 |
| | Mean Abs Dev | 0.023 | Mean Abs Dev | 0.032 |
| | -LogLikelihood | -51.817 | -LogLikelihood | -8.978 |
| | SSE | 0.019 | SSE | 0.008 |
| | Sum Freq | 24.000 | Sum Freq | 5.000 |

Table C2 shows the training and validation $R^2$ (how well training data fits with the model outcome), root average squared error (RASE). The model is overfit if the validation set has a RASE value that is higher than the training set (Validation RASE > Training RASE) and a lower $R^2$ value on the validation set (Validation $R^2$ < Training $R^2$). Mean Absolute (Abs) Deviation (Dev) is the average of the absolute values of the differences between the response and the predicted response. When the response is nominal or ordinal, the differences are between 1 and p (the fitted probability for the response level that occurred). Smaller values of the -Loglikelihood indicates better model fits. Sum of squared errors (SSE). Sum Frequency (Freq) gives the number of observations that are used.

**Table C3**. Tukey's HSD mean comparison between model for a glycan profile overview

| | Level | - Level | Difference | Std Err Dif | Lower CL | Upper CL | p-Value[1] |
|---|---|---|---|---|---|---|---|
| G0F | Kinetic | Exp | 0.0898 | 0.0473 | -0.0350 | 0.2147 | 0.2391 |
| | Kinetic | OPLS | 0.0579 | 0.0473 | -0.0669 | 0.1827 | 0.6147 |
| | Kinetic | Neural | 0.0523 | 0.0497 | -0.0786 | 0.1832 | 0.7185 |
| | Neural | Exp | 0.0375 | 0.0497 | -0.0934 | 0.1684 | 0.874 |
| | OPLS | Exp | 0.0319 | 0.0473 | -0.0929 | 0.1568 | 0.9062 |
| | Neural | OPLS | 0.0056 | 0.0497 | -0.1254 | 0.1365 | 0.9995 |
| G1F | Neural | Kinetic | 0.1035 | 0.0485 | -0.0246 | 0.2316 | 0.1542 |
| | Neural | OPLS | 0.0748 | 0.0485 | -0.0533 | 0.2029 | 0.4196 |
| | Exp | Kinetic | 0.0632 | 0.0434 | -0.0513 | 0.1778 | 0.4694 |

| | | | | | | | |
|---|---|---|---|---|---|---|---|
| | Neural | Exp | 0.0402 | 0.0485 | -0.0879 | 0.1683 | 0.8403 |
| | Exp | OPLS | 0.0345 | 0.0434 | -0.0800 | 0.1491 | 0.8561 |
| | OPLS | Kinetic | 0.0287 | 0.0434 | -0.0859 | 0.1433 | 0.9112 |
| G2F | Neural | Kinetic | 0.0184 | 0.0058 | 0.0030 | 0.0338 | 0.0133 |
| | OPLS | Kinetic | 0.0175 | 0.0058 | 0.0021 | 0.0329 | 0.0201 |
| | Exp | Kinetic | 0.0132 | 0.0058 | -0.0022 | 0.0286 | 0.1157 |
| | Neural | Exp | 0.0052 | 0.0058 | -0.0102 | 0.0206 | 0.8023 |
| | OPLS | Exp | 0.0043 | 0.0058 | -0.0111 | 0.0197 | 0.8775 |
| | Neural | OPLS | 0.0009 | 0.0058 | -0.0145 | 0.0163 | 0.9986 |

[1]A p-Value ≤ 0.05 is considered significant between the compared 2 groups, either model-model or model-experimental results.

In Table C3, the difference indicates the estimated difference between the means. Std Err Dif is the standard error of the difference. CL is the confidence interval for the difference in means for the lower and upper limits.

Table C3 is an overview comparison perspective between the models and experimental results shows that there is no significant difference by Tukey's HSD mean comparison (shown in Table 5). Slight differences can be observed for G2F between the kinetic model with the neural and OPLS model. Furthermore, p-value for kinetic model vs. experimental results were lower in value when examining the OPLS or the neural network model p-value with the experimental results. Across the board, both the OPLS and neural network model, the overall p-value was decreasing from G0F (average of the two p-value of 0.89) to G2F (average of the two p-value of 0.8).

**Table C4**. Trend depiction for UDP-Gal feed and consumption

| | Exp. | NN | OPLS | Kinetic |
|---|---|---|---|---|
| **qp of UDP-Gal** $_{(96\text{-}> 72\ hr)}$ | 0.002004 | 0.005918 | 0.021544 | 0.000506 |
| **% Diff (from Exp.) UDP-Gal** $_{(96\text{-}> 72\ hr)}$ | NA | 195.2471 | 974.8774 | -74.7622 |
| **qp of UDP-Gal** $_{(144\text{-}>118\ hr)}$ | 0.000776 | 0.002215 | 0.007708 | 0.000593 |
| **% Diff (from Exp.) UDP-Gal** $_{(144\text{-}>118\ hr)}$ | NA | 185.4432 | 893.1824 | -23.6165 |
| **1st Feed Absolute Difference** $_{(96\text{-}> 60\ hr)}$ | 0.442826 | 0.17216 | 0.17216 | 0.202682 |

| | | | | |
|---|---|---|---|---|
| **% Diff (from Exp.)** **(96 -> 60 hr)** | NA | 38.87748 | 38.87748 | 45.77012 |
| **2nd Feed Absolute Difference** **(144 -> 96 hr)** | 0.23653 | 0.062508 | 0.202682 | 0.381962 |
| **% Diff (from Exp.)** **(144 -> 96 hr)** | NA | 26.42724 | 85.68976 | 161.4856 |

Table C4 compares the specific consumption (qp) changes of UDP-Gal for 72 to 92 hours and 118 to 144 hours. This value indicates the biological consumption and production of model prediction for UDP-Gal. Furthermore, a % difference value is calculated based on the experimental dataset; ideally the % difference should be ±0% for exact model predictions on the experimental values. In addition, an absolute difference for the UDP-Gal concentrations were calculated during the 1$^{st}$ feed duration (60 to 96 hours) and 2$^{nd}$ feed duration (96 to 144 hours). The absolute difference indicates a significance in possible trends that can be observed in terms of predicting just UDP-Gal without the basis of cellular growth/death.

From Table C4, kinetic model had the best representation for qp of UDP-Gal. Although, NN and OPLS models both predicted the trends of UDP-Gal concentrations a lot better than the kinetic model. The significance in this result indicates that the kinetic model is only sufficient to obtain an overall UDP-Gal concentration; whereas NN and OPLS models can be more robust and capture more dynamic changes based on feeding strategy.
