# Supplementary figures and images for: Soft-sensor model development for CHO growth/production, intracellular metabolite, and glycan predictions
Source: Front Mol Biosci. 2024 Oct 22;11:1441885. doi: 10.3389/fmolb.2024.1441885 (PMC11535473; doi:10.3389/fmolb.2024.1441885)

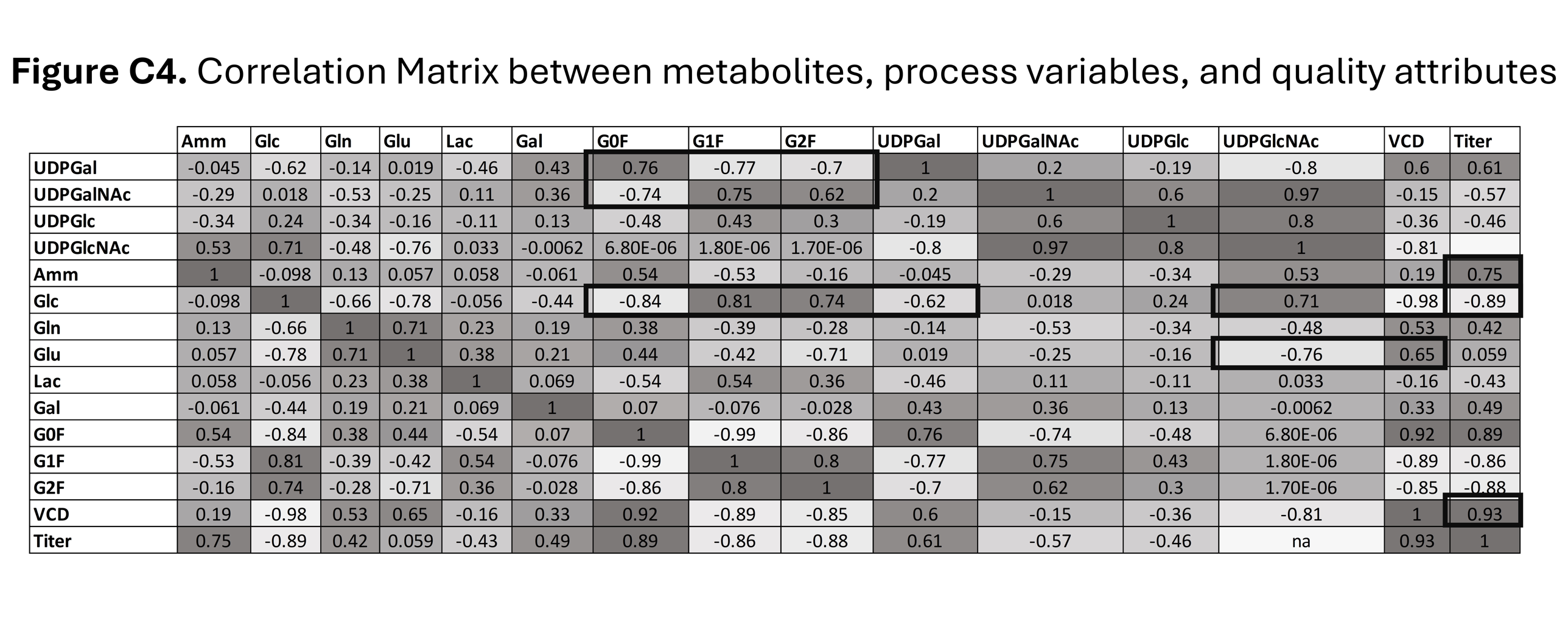

Supplement: Supplementary file 2 [file DataSheet2.zip › Supplementary Figures/Appendix_Figure C4.JPEG]

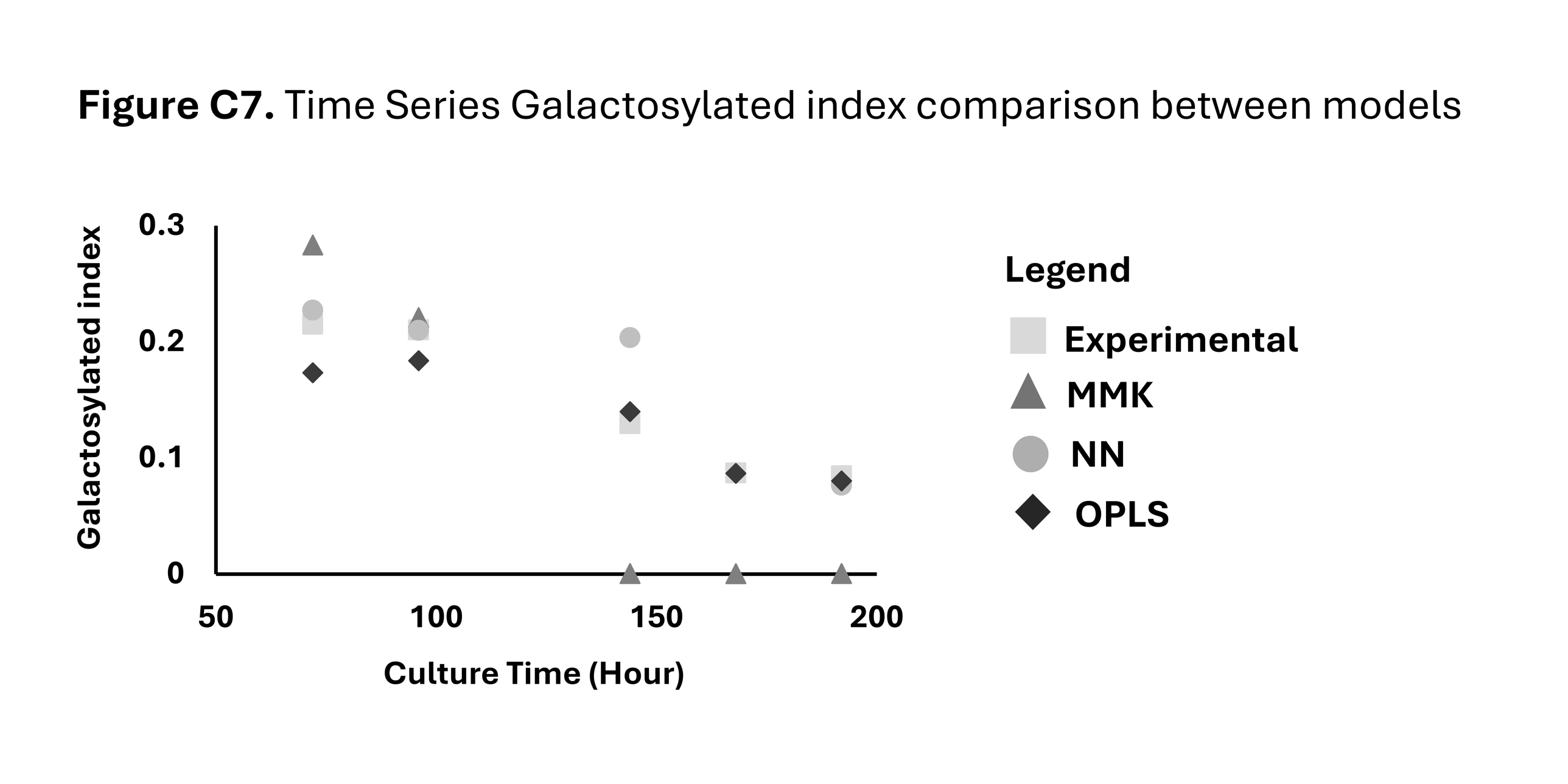

Supplement: Supplementary file 2 [file DataSheet2.zip › Supplementary Figures/Appendix_Figure C7.jpg]
